# Effects of the Antidepressant Amitriptyline on Juvenile Brown Trout and Their Modulation by Microplastics

**DOI:** 10.3390/toxics10120763

**Published:** 2022-12-07

**Authors:** Hannah Schmieg, Stefanie Krais, Kathrin Kübler, Aki S. Ruhl, Isabelle M. Schmidgall, Christian Zwiener, Heinz-R. Köhler, Rita Triebskorn

**Affiliations:** 1 Animal Physiological Ecology, University of Tübingen, Auf der Morgenstelle 5, 72076 Tübingen, Germany; 2 Water Treatment, Technische Universität Berlin, KF 4, Str. des 17. Juni 135, 10623 Berlin, Germany; 3 German Environment Agency (UBA), Section II 3.3 (Water Treatment), Schichauweg 58, 12307 Berlin, Germany; 4 Environmental Analytical Chemistry, University of Tübingen, Schnarrenbergstr. 94–96, 72076 Tübingen, Germany; 5 Steinbeis Transfer Center for Ecotoxicology and Ecophysiology, Blumenstr. 13, 72108 Rottenburg, Germany

**Keywords:** microplastics, polystyrene, antidepressant, amitriptyline, brown trout, behavior, oxidative stress, acetylcholinesterase, cortisol

## Abstract

Pharmaceuticals such as antidepressants are designed to be bioactive at low concentrations. According to their mode of action, they can also influence non-target organisms due to the phylogenetic conservation of molecular targets. In addition to the pollution by environmental chemicals, the topic of microplastics (MP) in the aquatic environment came into the focus of scientific and public interest. The aim of the present study was to investigate the influence of the antidepressant amitriptyline in the presence and absence of irregularly shaped polystyrene MP as well as the effects of MP alone on juvenile brown trout (*Salmo trutta* f. *fario*). Fish were exposed to different concentrations of amitriptyline (nominal concentrations between 1 and 1000 µg/L) and two concentrations of MP (10^4^ and 10^5^ particles/L; <50 µm) for three weeks. Tissue cortisol concentration, oxidative stress, and the activity of two carboxylesterases and of acetylcholinesterase were assessed. Furthermore, the swimming behavior was analyzed in situations with different stress levels. Exposure to amitriptyline altered the behavior and increased the activity of acetylcholinesterase. Moreover, nominal amitriptyline concentrations above 300 µg/L caused severe acute adverse effects in fish. MP alone did not affect any of the investigated endpoints. Co-exposure caused largely similar effects such as the exposure to solely amitriptyline. However, the effect of amitriptyline on the swimming behavior during the experiment was alleviated by the higher MP concentration.

## 1. Introduction

Depression is a common chronic disease in humans and the number of reported cases has increased dramatically worldwide in the last 30 years [1]. Consequently, the consumption of antidepressants in OECD (Organization for Economic Co-operation and Development) countries doubled between 2000 and 2017 [2]. Tricyclic antidepressants which are non-selective monoamine reuptake inhibitors were among the first pharmaceuticals used to treat depression [3,4]. Amitriptyline is the most prescribed tricyclic antidepressant and the fifth most used antidepressant in total in Germany with a prescription rate of 80 million defined daily doses in 2018 [4,5]. In addition to treating depression, amitriptyline is used for migraine prophylaxis and the treatment of chronic pain [4]. The mode of action of amitriptyline is the inhibition of the reuptake of serotonin, noradrenaline, and—considerably less effectively—dopamine, thereby increasing the time of the respective neurotransmitters in the synaptic cleft [6]. Interactions of amitriptyline with other receptors are also known, for example with muscarinic acetylcholine receptors [7], histamine receptors [8], or neurotrophic tyrosine kinase A/B receptors [9].

Residues of pharmaceuticals and their metabolites enter the environment mainly via wastewater and are regularly detected in surface waters worldwide [10,11]. In effluents of wastewater treatment plants in the UK, concentrations up to 357 ng/L amitriptyline have been measured [12]. The antidepressant can also be detected worldwide in lakes and rivers in concentrations of up to 71 ng/L [13,14,15,16,17,18]. In a Brazil river basin amitriptyline concentrations up to 196 ng/L were reported [19]. Furthermore, residues of the tricyclic antidepressant were detected in groundwater in China [20] and even in drinking water in France [21].

Effects of pharmaceuticals in aquatic non-target organisms are likely since pharmaceuticals are often rather stable in the environment and bioactive in low concentrations [10,11,22]. Furthermore, molecular targets for pharmaceuticals are present in many species [23]. Like in humans, amitriptyline was shown to influence serotonin, noradrenaline, and dopamine levels in the brain of adult zebrafish (*Danio rerio*) [24,25]. Additionally, changes in behavior have been observed in zebrafish and brown trout (*Salmo trutta* f. *fario*) exposed to the antidepressant: the fish showed altered resting behavior, reduced swimming activity covering less distance, and even ataxic behavior such as looping. Furthermore, swimming on the side or in a vertical position occurred in amitriptyline-exposed fish [24,25,26,27,28]. In addition to these effects, the antidepressant was shown to affect the growth and development of zebrafish [28,29], common carp (*Cyprinus carpio*) [30], and brown trout [27]. Moreover, amitriptyline has been reported to modulate the immune response of fish [29,31], affecting their oxidative stress level [29,30,32] and influencing their acetylcholinesterase activity [27]. Most of these studies have been performed with zebrafish and common carp, while little is known about possible effects in other fish species. Furthermore, the studies investigated predominantly effects in vitro or on early life stages while data on other life stages are limited.

In addition to dissolved chemicals, microplastics (MP) as a potential environmental stressor has moved into focus of scientific and public interest in recent years. MP are detected ubiquitously in the environment, even in areas without apparent anthropogenic influence (e.g., [33,34,35,36,37]). According to Li et al. [38] concentrations between 0.00012 and 2867 particles/L have been reported in surface waters worldwide. Without further strategies to reduce plastic emissions, the annual rate of MP entering the environment is assumed to increase during the next decades [39].

The environmental risk related to MP is a matter of discussion since many factors such as polymer type, size, age, shape, quantity, and quality of additives influence their effects on organisms. Furthermore, the possible interactions of MP with other contaminants and the different susceptibility of different feeding types of exposed organisms are of crucial relevance for potential effects and increase the uncertainty of risk assessment for MP. Mechanical effects of MP can lead to anorexia in fish [40], to oxidative stress or inflammation (e.g., [41,42,43]). Current opinion assigns most environmental relevance to small MP or even nano-sized particles, as they can be taken up by cells [44,45]. Moreover, MP have also been considered a source of soluble contaminants since additives or residual monomers can leak from the particles [46,47]. Furthermore, MP have been shown to amplify the effects of dissolved environmental chemicals, e.g., paraquat [48], butylated hydroxyanisole [49], or pyrene [50]. On the other hand, the sorption of pollutants onto or within MP can also reduce the bioavailability of chemicals, which then can lead to reduced effects on the environment [51,52,53].

By the usual means, the determined environmental risk of MP is classified as rather low at present [54,55,56]. However, long-term ecotoxicological experiments as well as data on feral freshwater organisms with irregularly shaped MP are still rare and interference of MP with other chemicals is still not fully understood [54,55]. Thus, experiments addressing these gaps of knowledge are urgently needed to decrease the uncertainty in the environmental risk assessment of MP.

On this background, the aim of the present study was to investigate effects from the biochemical to the individual level in juvenile brown trout after exposure to different amitriptyline and MP concentrations and to reveal whether effects induced by amitriptyline are influenced by co-exposure with MP.

## 2. Materials and Methods

### 2.1. Test Organism

Juvenile brown trout (*Salmo trutta* f. *fario*) were purchased from a commercial trout farm (Forellenzucht Lohmühle, Alpirsbach-Ehlenbogen, Germany). The breeding facility is regularly controlled and classified as category I, disease-free (Council Directive 2006/88/EC) [57]. Fish were acclimatized to lab conditions for at least one week before the experiments.

### 2.2. Test Substance

Amitriptyline hydrochloride was purchased from Sigma Aldrich (CAS number: 549-18-8; chemical formula: C_20_H_23_N · HCl). All given amitriptyline concentrations refer to the pure amitriptyline portion. No organic solvent was used in both experiments. For the first experiment two stock solutions were prepared with bi-distilled water: stock solution I contained 100 mg/L amitriptyline and for stock solution II, stock solution I was diluted to a concentration of 1 mg/L. For the second experiment, a stock solution with a concentration of 25 mg/L amitriptyline was prepared.

Irregularly shaped PS MP were produced according to the methods of Eitzen, et al. [58]. Transparent PS pellets were purchased (Polystyrol 158 K, BASF, Ludwigshafen, Germany, density 1.05 g/mL) and cryogenically milled (CryoMill, Retsch, Haan, Germany). The PS MP were suspended in ultra-pure water without adding any surfactant. The particle concentration (56,240 particles/mL) in the pure suspension was determined with a particle counter (SVSS, PAMAS, Rutesheim, Germany) by light extinction in a laser-diode sensor (type HCB-LD-50/50). The size distribution of the particles is given in Table S1 and Figure S1 in the Supplementary Materials. For more information on the PS particles see Schmieg et al. [27] and Schmieg et al. [59]. The particles were fractionated to a nominal size below 50 µm with a micro-sieve (polyamide monofilament). The smallest and therefore possibly biologically relevant particles took the largest share in terms of number.

### 2.3. Experiments with Juvenile Trout

The experiments were conducted in two runs. Brown trout were exposed to defined amitriptyline and particle concentrations in a semi-static three-block design (triplicates) for three weeks in each run. Experiments took place in a thermostat-controlled chamber set to 7 °C and a light/dark cycle of 10/14 h. Glass aquaria with 15 L test media were placed in randomized order in each block to account for potential confounding factors. Each tank was equipped with a glass pipette connected to compressed air via silicone tubes for aeration and shaded from direct light. Per tank, 10 fish were exposed (thus 30 fish per treatment group). Trout were fed daily approximately 3% of their body weight (0.8 mm size, Incio Plus, Biomar, Brande, Denmark). A total of 50% of the water was exchanged twice a week. Amitriptyline stock solutions were freshly prepared immediately before the water exchanges. Vials containing MP stock suspensions were rinsed four times to avoid incomplete transfer of particles. To adjust the final amitriptyline and MP concentrations, stock solutions and stock suspensions were diluted with filtered and aerated tap water (after pretreatment with iron filter, particle filter, and activated carbon filter; Filwatec, Bad Liebenzell, Germany). Mortality and abnormal behavior were recorded daily, and dead fish were removed immediately.

**Figure 1:**
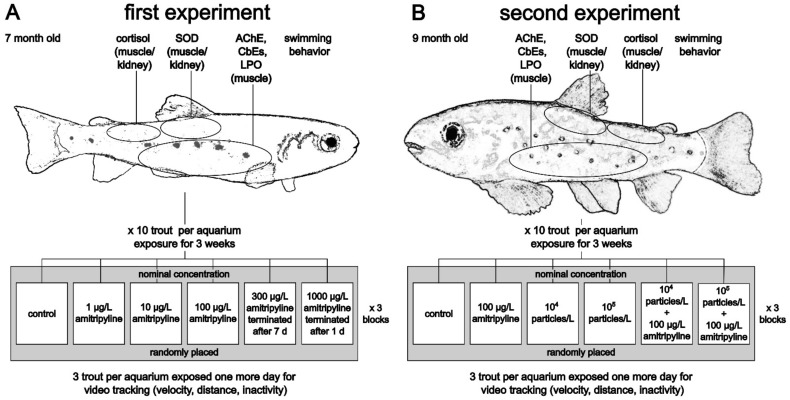
Overview of the experimental designs of the first (**A**) and second (**B**) experiment. Grey boxes indicate one block (SOD: superoxide dismutase; AChE: acetylcholinesterase; CbEs: carboxylesterases; LPO: lipid peroxidation).

In the first experiment, 7 months old trout were exposed to nominal 0, 1, 10, 100, 300, and 1000 µg/L amitriptyline (Figure 1A). Fish exposed to nominal 300 µg/L and 1000 µg/L showed severe effects and had to be euthanized after 7 d and 24 h, respectively. At the beginning and the end of the experiment physico-chemical water parameters were recorded (average values: pH = 7.5 ± 0.2, temperature = 6.6 ± 0.3 °C, conductivity = 481.5 ± 3.1 µS/cm, oxygen concentration = 11.2 ± 0.1 mg/L, oxygen saturation = 96 ± 1%; see Supplementary Table S2). In all aquaria, nitrite (NO_2_^−^) concentration did not exceed 0.1 mg/L.

In the second experiment, effects of amitriptyline and MP were investigated in 9-month-old trout (Figure 1B). In addition to a control group, fish were exposed to nominal 100 µg/L amitriptyline, 10^4^ particles/L PS MP (MP_tt_), 10^5^ particles/L PS MP (MP_ht_) and the mixtures 100 µg/L amitriptyline + 10^4^ particles/L (MIX_tt_), and 100 µg/L amitriptyline + 10^5^ particles/L (MIX_ht_). The amitriptyline concentration was chosen, since the nominal concentration of 100 µg/L caused effects in brown trout in the first experiment, but no concerns for animal welfare. The PS MP concentrations were chosen based on used particle concentrations in other exposure studies with MP. Physico-chemical water parameters were measured at the beginning and the end of the experiment (average values: pH = 7.6 ± 0.1, temperature = 6.4 ± 0.2 °C, conductivity = 480.2 ± 4.7 µS/cm, oxygen concentration = 11.2 ± 0.3 mg/L, oxygen saturation = 95 ± 2%, total hardness = 15.1 ± 0.5; see Supplementary Table S3). Measured NO_2_^−^ concentrations were equal to or less than 0.1 mg/L.

At the end of the experiments, fish were euthanized by an overdose of 1 g/L MS222 (tricaine methanesulfonate) adjusted to pH 7 with NaHCO_3_, and death was guaranteed by severance of the spine. Length and weight of each animal were measured. Fish were dissected, and samples for lipid peroxidation (LPO; muscle), superoxide dismutase (SOD; muscle/kidney), cortisol (muscle/kidney), acetylcholinesterase (AChE; muscle), and carboxylesterases (CbE; muscle) quantification were immediately frozen in liquid nitrogen and stored at −80 °C until further analyses (Figure 1). Samples for determination of SOD activity were rinsed in phosphate-buffered saline (pH 7.4) before freezing.

In both experiments, three remaining fish per tank were continuously exposed and used one day later for quantitative assessment of their swimming behavior by a video tracking system (described in Section 2.6) and dissected afterward.

### 2.4. Chemical Analyses

Water samples were taken at the beginning and the end of both experiments as well as before and after a water exchange. Water samples of the three replicates of each treatment group were pooled and stored (~2.5 years) at −20 °C prior to analyses. The concentrations of amitriptyline were determined with a 1290 Infinity HPLC system (Agilent Technologies, Waldbronn, Germany) and a triple quadrupole mass spectrometer (MS, 6490 iFunnel Triple Quadrupole LC/MS, Agilent Technologies) in ESI (+) mode. For separation, an Agilent Poroshell-120-EC-C18 (2.7 µm, 2.1 × 100 mm) column at a flow rate of 0.4 mL/min was applied. The column temperature was maintained at 40 °C. Eluent A was water (containing 0.1% formic acid) and eluent B was acetonitrile (containing 0.1% formic acid). Gradient elution was used: 0–1 min 5% B, linear increase to 100% B within 7 min, hold for 7 min at 100% B. After switching back to the starting conditions, reconditioning time of 3 min was employed. Samples were kept in the autosampler at 10 °C, the injection volume was 10 µL. Samples were measured undiluted or after a 50-fold dilution. The detection limit of amitriptyline (mass transition *m*/*z* 278.2 → 117.1) was 100 ng/L. More details on operating parameters of the triple quadrupole MS are listed in the (Supplementary Tables S4 and S5). For calculating mean exposure concentrations values below the limit of quantification (LoQ) were substituted with LoQ/√2. Despite the recommendation of the OECD test guideline no. 203 [60], nominal concentrations are used to report the results, due to uncertainties concerning the amitriptyline concentrations.

### 2.5. Behavior during Exposure

The position of the fish in the tank (upper or lower half of the aquaria) was recorded daily from the second day on in the first experiment and from the sixth day on in the second experiment. For that purpose, the covers of the exposure tanks were removed, and a white sheet of paper was placed behind the aquaria to improve contrast. After 5 min of acclimatization to the light conditions, 3 pictures (5 min between each) were taken with a Panasonic DMC-TZ56 camera. Each image was visually assessed with regard to the number of individuals present in the upper and lower half of the tanks. Subsequently, the data recorded for each individual tank on the same day were averaged.

### 2.6. Video Tracking of Swimming Behavior

For video tracking of the swimming behavior at the end of the experiments, three fish of each tank were transferred into small aquaria (17.0 cm × 17.0 cm × 8.5 cm) filled with one liter of the corresponding test medium. Four aquaria were recorded simultaneously. Each aquarium was equipped with a camera (Basler acA 1300-60 gm, 1.3-megapixel resolution, Basler AG, Ahrensburg, Germany, lens: 4.5–12.5 mm; 1:1.2; IR 1/2″) fixed 32 cm above the water surface. Subsequently, the four aquaria in their entirety were surrounded with white polystyrene plates. Four lamps (2700 K, 1521 lm each) were placed inside the enclosure facing the top plate to provide indirect illumination. The swimming behavior was recorded for 20 min whereby the first 2 min were regarded as acclimatization phase and therefore not analyzed. In the remaining 18 min of recordings, trout were individually center-point tracked and the total distance moved, the average velocity, and the time of no movement was analyzed with EthoVision 12 XT (Noldus Information Technology bv, Wageningen, Netherlands). To avoid failures of automated tracking, for example, swaps between the individuals, the whole footage was sighted afterward and manually corrected if necessary.

### 2.7. AChE and CbE Activity

To determine the acetylcholinesterase (AChE) and carboxylesterase (CbE) activities, muscle tissue was homogenized with TRIS buffer (20 mM TRIS_base_, 20 mM NaCl, inhibitor mix, pH 7.3) in a ratio of 1:5 (*w*/*v*). Following, the samples were centrifuged (5000 rcf, 10 min, 4 °C) and 50% glycerol (1/4 of the volume of the supernatant) was added to the supernatant. Samples were stored at −20 °C until the final analysis. To determine the total protein content in the samples, the method of Lowry et al. [61] modified by Markwell et al. [62] was used. AChE activity was analyzed spectrophotometrically according to the method of Ellman et al. [63] modified by Rault et al. [64]. The method described by Sanchez-Hernandez et al. [65] was used to measure the activity of two CbEs with the substrates 5 mM 4-nitrophenyl acetate (NPA) and 5 mM 4-nitrophenyl valerate (NPV). In all plates, the absorbance was measured at 405 nm (Bio-Tek Instruments, Winooski VT, USA) and all samples were analyzed in triplicates. The specific activities of the enzymes are given per mg protein content. One micromole substrate hydrolyzed per min has been defined as one unit.

### 2.8. Cortisol Level

The cortisol level was measured in selected muscle/kidney samples with the commercially available fish cortisol ELISA Kit by Cusabio Technology LCC (Huston, Texas, USA). From the first experiment, tissue samples of 8 fish per replica (24 per treatment) of all treatment groups were analyzed. From the second experiment, 7–8 fish per replica were analyzed from each group. Since no effect occurred in MP_ht_ and MIX_ht_, the lowest MP group (MP_tt_) and the co-exposure of amitriptyline and the lowest MP group (MIX_tt_) were not analyzed. The tissue samples were homogenized in phosphate-buffered saline (1:11 *w*/*v*). Subsequently, after two freeze–thaw cycles to break the cell membranes, samples were centrifuged (5000 rcf, 5 min, 4 °C). Prior to the analysis, supernatants were stored at −20 °C. For the assay, supernatants were diluted (1:10 *v*/*v*) with the sample buffer included in the kit. In the pre-coated 96-well plate 50 µL antibody (specific for cortisol and horseradish peroxidase (HRP) conjugated goat anti-rabbit antibody) and 50 µL of either standard or sample was pipetted. Samples were analyzed in duplicates. In the following, the plates were incubated at 37 °C for 40 min, and subsequently, 3 washing cycles were performed. An amount of 100 µL HRP-conjugate was added to each well and the plate was incubated for another 30 min at 37 °C. After 5 washing cycles, 90 µL 3,30,5,50-Tetramethyl [1,10-biphenyl]-4,40-diamine substrate was added, and the plates were incubated for 20 min at 37 °C. After that, the reaction was stopped with 50 µL of a stopping solution, and photometrical measurements were conducted at 450 nm and 570 nm for wavelength correction.

### 2.9. Lipid Peroxide Level

The lipid peroxide content was determined with the ferrous oxidation xylenol orange (FOX) assay according to Hermes-Lima et al. [66] and Monserrat et al. [67] and adjusted for 96-well plates. Muscle samples were homogenized with HPLC-grade methanol (tissue to methanol ratio 1:6 *w*/*v*) and centrifuged (15,000 rcf, 5 min, 4 °C). In a pretest, sample volume and incubation time were adjusted for optimal results. Subsequently, 50 µL of 0.75 mM FeSO_4_, 75 mM H_2_SO_4_, and 0.3 mM xylenol orange solution, 30 µL, and 40 µL supernatant (for the first experiment and second experiment, respectively), were added in each well of a 96-well plate. Samples were analyzed in triplicates. To consider potential Fe in the samples, a sample blank in which the FeSO_4_ solution was replaced by bi-distilled water was performed. In the following, bi-distilled water was used to adjust the volume of each well to 200 µL. Plates were incubated for 165 min and 180 min (for the first experiment and second experiment, respectively). After incubation, the absorbance at 570 nm (ABS570) was measured in an automated plate reader (Bio-Tek Instruments, Winooski VT, USA). Afterward, 1 µL of 1 mM cumene hydroperoxide (CHP) solution was added to each well. The plates were incubated for 30 min at room temperature and the absorbance at 570 nm was measured for a second time. Sample values were corrected with the corresponding sample blanks. CHP equivalents per mg wet weight (CHPequiv.) were calculated using the following equation:

(1) $$CHPequiv.=\frac{ABS570\;sample\;}{ABS570\;sample\;and\;CHP\;}*CHP\;\left(1\;µL\right)*\frac{total\;volume\;in\;well\;\left(200\;µL\right)}{sample\;volume}*dilution\;factor$$

### 2.10. Activity of Superoxide Dismutase

Tissue samples (muscle/kidney) were homogenized in 20 mM HEPES buffer (1 mM EGTA, 210 mM mannitol, and 70 mM sucrose, pH 7.2) in a ratio of 1:5 (*w*/*v*). Subsequently, samples were centrifuged (1500 rcf, 4 °C, 5 min) and the supernatant was stored at −80 °C until further analyses. Superoxide dismutase activity was analyzed with the Cayman Chemical superoxide dismutase assay kit (item no. 706002, Cayman Chemical, Ann Arbor, MI, USA). Prior to the assay, the supernatant was diluted 5:150 (*v*/*v*) with 50 mM TRIS-HCl buffer (pH 8.0). In this assay kit, tetrazolium salt is utilized to assess superoxide radicals generated by hypoxanthine and xanthine oxidase. Superoxide dismutase (SOD) reduces the amount of superoxide radicals. The assay can detect Cu/Zn-SOD, Mn-SOD, and Fe-SOD. Absorbance was measured at 450 nm (Bio-Tek Instruments, Winooski, VT, USA). All samples were analyzed in duplicates.

### 2.11. Statistical Analysis

For statistical purposes, the software R (version 3.6.2; [68]) was used. Due to their different exposure times (earlier termination for animal welfare reasons), the treatment groups with nominal 300 µg/L and nominal 1000 µg/L amitriptyline in the first experiment were not included in the statistical analyses. The α-level was set to 0.05. To analyze the data on mortality, a mixed-effect Cox model (package coxme; [69]) with treatment as fixed effect and aquarium ID as random effect was used. Data for length, weight (second experiment), AChE, CbE-NPA, CbE-NPV, SOD, velocity at last minute of recording (first experiment) and total distance moved were analyzed with a linear mixed model (package lme4; [70]) with treatment as fixed and aquarium ID as random effect. For the analysis of the cortisol level in the second experiment, “inclusion of fish in video tracking” was used as an additional fixed effect, since potential stress caused by the video tracking could influence the cortisol level. To analyze the mean velocity and time of no movement during the video tracking, recording time beside treatment was included as a fixed factor. If necessary, data were transformed to gain normal distribution and homogeneity of variances (see Supplementary Table S6). Data for weight (first experiment) could not be transformed to homogeneity of variances, so a Welch-ANOVA was performed. Data of the cortisol level and time of no movement at last minute of the recording in the first experiment and of LPO level in both experiments were not normally distributed. Therefore, these endpoints were analyzed with a Kruskal–Wallis test and Conover’s many-to-one post hoc test, if significant. Multiple comparisons were corrected using the method of Benjamini and Hochberg [71]. The position of the fish in the aquaria during the experiments were analyzed with generalized linear mixed models with treatment group and days of exposure as fixed factors and aquarium ID as random factor. In the second experiment the package glmmTMB [72] was used to handle zero inflated data since no fish of the control group, MP_tt_ and MP_ht_ stayed in the upper half of the aquaria. In the first experiment, Dunnett’s test was used as a post hoc test to compare the treatment groups with the control group whereas in the second experiment Tukey’s all-pair comparisons were applied to be able to compare results of all groups (Table S7).

### 2.12. Animal Welfare

All experiments were performed according to German legislation and were approved by the regional council of Tübingen, Germany (authorization number ZO 2/16).

### 2.13. Credibility of Data

Moermond et al. [73] proposed criteria for reporting and evaluation of ecotoxicity data (CRED). Details on the fulfillment of these criteria are provided in the Supplementary Materials.

## 3. Results

### 3.1. Amitriptyline Concentrations

In both experiments, no amitriptyline was detected in the control groups or treatment groups that solely contained MP at any investigated time point. In the first experiment, the measured amitriptyline concentrations at the start of the experiment were only between 8% and 14% of the nominal concentration (Table 1). The concentrations of the groups which contained nominally 300 µg/L and 10 µg/L were relatively consistent at the different time points. In contrast, concentrations of the two treatment groups with the nominal level of 100 µg/L and 1 µg/L dropped under the limit of quantification (LoQ: 0.1 µg/L).

In the second experiment, the measured amitriptyline concentrations were between 5.7 µg/L and below the limit of quantification, i.e., 0.1 µg/L (Table 2). At the end of the experiment, the amitriptyline concentrations of the three groups with amitriptyline were between 0.6 µg/L and 0.1 µg/L. To understand the reasons for the discrepancy of nominal and measured amitriptyline concentrations and to exclude storage affected the measured concentrations samples of the stock solutions were analyzed. In the stock solution with nominal 1000 µg/L amitriptyline the measured amitriptyline concentration was 209 µg/L whereas in the stock solution with nominal 25,000 µg/L the detected amitriptyline concentration was 2545 µg/L (circa 21% and 10% of the nominal concentrations). Due to the uncertainties concerning the amitriptyline concentrations in both experiments, nominal concentrations were used to report results.

### 3.2. Mortality and Biometric Values

At the end of the first experiment, fish were on average, 6.1 ± 0.6 cm long and weighed 2.2 ± 0.7 g (Table 3). The two months older fish in the second experiment had a length and weight of 7.0 ± 0.7 cm and 3.1 ± 0.9 g (Table 4). The overall mortality rate was 17.5% in the first experiment while no fish died during the second experiment (Table 3 and Table 4). No significant differences in the mortality rate, the length, and the body mass of trout between all treatment groups occurred in both experiments (first experiment: mortality *Χ^2^* = 1.3398, *df* = 3, *p* = 0.720; weight *F* = 1.4473, *df* = 3/50.03, *p* = 0.240; length *F* = 0.0809, *df* = 3/95, *p* = 0.970; second experiment: weight *F* = 2.0216, *df* = 5/173, *p* = 0.078; length *F* = 1.4296, *df* = 5/173, *p* = 0.216). The nominal concentration of 1000 µg/L amitriptyline caused severe effects on behavior and respiration within one day of exposure. For animal welfare, the treatment group was terminated immediately after the occurrence of these effects. Fish of the treatment group with a nominal concentration of 300 µg/L amitriptyline had to be euthanized after one week since behavioral effects met termination criteria (see Table S8 for further results of the two treatment groups).

### 3.3. Behavior during Exposure

Trout exposed to a nominal 300 µg/L of amitriptyline showed ataxic movements such as side or vertically swimming. This behavior intensified with increasing exposure time. For animal welfare reasons fish in this group were euthanized after one week. Fish exposed to nominal 100 µg/L amitriptyline showed also abnormal behavior such as swimming upside down, on the side, or vertically. However, this behavior always only occurred for a short time which is why the test did not have to be terminated earlier. In the second experiment, the described behavior was observed sporadically in fish exposed to amitriptyline and in the MIX_tt_ group.

**Figure 2:**
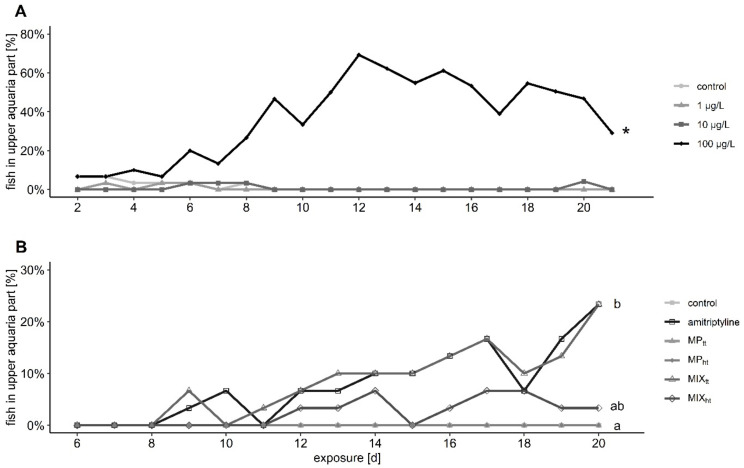
Percentage of fish present in the upper half of the aquaria during the exposure to different amitriptyline concentrations in the first experiment (**A**) and the second experiment (**B**) to amitriptyline or 10^4^ particles/L PS MP (MP_tt_), 10^5^ particles/L PS MP (MP_ht_) or the mixtures amitriptyline + 10^4^ particles/L (MIX_tt_), and amitriptyline + 10^5^ particles/L (MIX_ht_). 2 (**A**): Significant differences towards the control group are indicated with an asterisk. 2 (**B**): Significant differences between the treatment groups are displayed with different letters. Further statistical information is provided in the Supplementary Table S7.

Trout in the control groups and the two groups with solely MP swam and rested mostly or exclusively in the lower part of the tanks during the entire experiment (Figure 2). Prolonged exposure to amitriptyline significantly increased the percentage of fish that stayed in the upper half of the aquaria. With a maximal 69% compared to a maximal 23% of fish in the upper half of the tank the effect on the behavior was considerably more pronounced in the first experiment than in the second. The effect on the position of fish in the aquaria was also observed in trout co-exposed to 10^4^ particles/L and amitriptyline (MIX_tt_). In contrast, fewer fish exposed to the mixture of amitriptyline and 10^5^ particles/L (MIX_ht_) swam in the upper part of the aquarium and no significant difference between this group and either amitriptyline treatment group or the control group occurred (first experiment: treatment: *F* = 19.066, *df* = 3; days of exposure: *F* = 34.248, *df* = 1; second experiment: treatment: *Χ^2^* = 30.037, *df* = 5, *p* < 0.001; days of exposure: *Χ^2^* = 14.320. *df* = 1, *p* < 0.001; amitriptyline/MIX_ht_ *p* = 0.190).

### 3.4. Video Tracking

**Figure 3:**
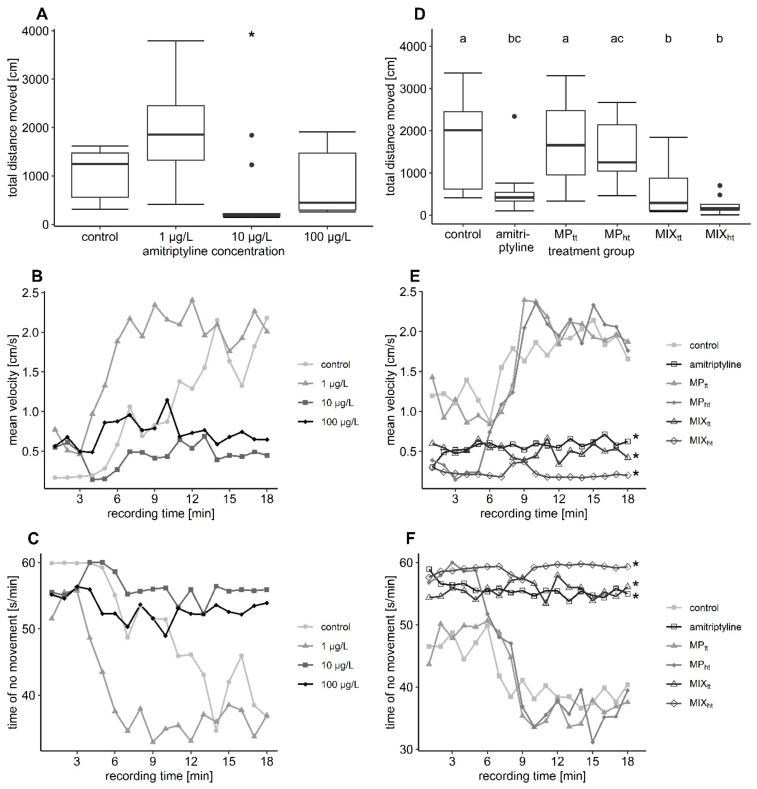
Results of the video tracking analysis. In plots (**A**–**C**) the results of the first experiment are depicted, in plots (**D**–**F**) the results of the second experiment (MP_tt_: 10^4^ particles/L PS MP; MP_ht_: 10^5^ particles/L PS MP; MIX_tt_: amitriptyline + 10^4^ particles/L; MIX_ht_: amitriptyline + 10^5^ particles/L). The boxplots display the median, the 25th, and 75th percentiles as well as minimum and maximum values (whiskers) whereas dots indicate outliers. Significant differences towards the control group (complete recording time) are indicated by asterisks. Significant differences between the treatment groups are indicated by different letters. 3 (**B**,**C**): Significant differences between the control group and 10 µg/L as well as 100 µg/L amitriptyline occurred at the end of recording time. Further statistical information is provided in Supplementary Table S7.

In the first experiment, the distance covered during the video tracking in the control group was 1101 ± 528 cm. The covered distance increased by 69% after exposure to nominal 1 µg/L amitriptyline and reached 1864 ± 1039 cm. In contrast, exposure to nominal 10 µg/L and to 100 µg/L amitriptyline reduced the covered distance by 56% and 29% to 486 ± 615 cm and 786 ± 652 cm, respectively (Table 3). However, data were very variable and therefore only the difference between the control group and the group treated with 10 µg/L was statistically significant (Figure 3A; first experiment: distance moved *F* = 4.904, *df* = 3/8, *p* = 0.032). In the second experiment, fish exposed to amitriptyline, or the two mixtures covered significantly less distance (between 65–86%) than the control group, while MP had no effect on the fish’s behavior (Figure 3D, Table 4; second experiment: distance moved *F* = 7.9203, *df* = 5/12.01, *p* = 0.002).

The mean velocity changed significantly over time in both experiments (mean velocity_recording time_: first experiment *F* = 84.6495, *df* = 1/635, *p* < 0.001; second experiment *F* = 72.6361. *df* = 1/953.11 *p* < 0.001). After a short phase with relatively low mean velocity, fish of the control groups swam with a relatively high velocity of 2.18 cm/s (first experiment) and 1.66 cm/s (second experiment) at the end of the video tracking (Figure 3B,E). The initial phase with lower velocity was considerably longer in the control group of the first than in the second experiment. Fish exposed to MP showed the same behavioral pattern as the control group. Fish exposed to amitriptyline in contrast swam with a comparatively low velocity during the whole recording. Despite the behavioral pattern of the control group being more similar to the behavior of fish exposed to amitriptyline in the first experiment, a trend towards reduced mean velocity in treatment groups with amitriptyline occurred over the complete recording time (mean velocity_treatment_: *F* = 3.8452, *df* = 3/8, *p* = 0.057). At the end of the recording, the mean velocity of the treatment groups with 10 and 100 µg/L amitriptyline was significantly reduced compared to the velocity of the control group (Table 3; *F* = 4.8169, *df* = 3/8, *p* = 0.034). In the second experiment trout exposed to amitriptyline or the mixture of amitriptyline and MP (MIX_tt_ and MIX_ht_) swam over the whole time span with a significantly reduced mean velocity compared to the control group (Table 4; mean velocity_treatment_: *F* = 6.2046, *df* = 5/12.43, *p* = 0.004).

Trout were relatively inactive at the start but moved significantly more over the recording (Figure 3C,F; no movement_recording time_: first experiment *F* = 76.6406, *df* = 1/635, *p* < 0.001; second experiment *F* = 70.3521, *df* = 1/952.92, *p* < 0.001). MP did not change the level of inactivity. Comparable to the mean velocity, fish exposed to amitriptyline in the first experiment showed only a trend towards a higher level of inactivity over the whole recording time (no movement_treatment_: *F* = 3.2578, *df* = 3/8, *p* = 0.081). Nevertheless, at the end of the video tracking, fish exposed to 10 µg/L and 100 µg/L amitriptyline moved significantly less compared to the control group (Table 3; *Χ^2^* = 15.923, *df* = 3, *p* = 0.001). In the second experiment, time spent in the inactivity of the amitriptyline treatment group, MIX_tt_, and MIX_ht_ was, compared to the control group, significantly higher over the complete recording time (Table 4; no movement_treatment_: *F* = 6.1867, *df* = 5/12.25, *p* = 0.004).

### 3.5. Biochemical Responses

**Figure 4:**
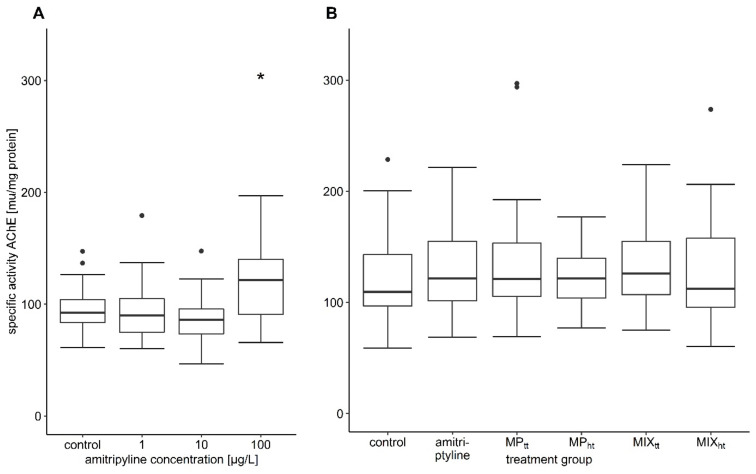
Specific activity of AChE. (**A**) first experiment: control *n* = 25, 1 µg/L and 100 µg/L amitriptyline *n* = 24, 10 µg/L amitriptyline *n* = 26; (**B**) second experiment: all treatment groups *n* = 30 (MP_tt_: 10^4^ particles/L PS MP; MP_ht_: 10^5^ particles/L PS MP; MIX_tt_: amitriptyline + 10^4^ particles/L; MIX_ht_: amitriptyline + 10^5^ particles/L). In the boxplots the median, the 25th, and 75th percentiles as well as minimum and maximum values (whiskers) are displayed; the dots indicate outliers. Significant difference compared to the control group is indicated with an asterisk.

The activity of AChE was significantly increased by 25% in fish exposed to a nominal 100 µg/L amitriptyline compared to the control group in the first experiment (Table 3; Figure 4A; *F* = 5.5384, *df* = 3/7.5527, *p* = 0.026). Trout exposed to 300 µg/L amitriptyline had also a considerably higher activity of AChE. However, the different exposure times of the treatment groups with 300 µg/L and 1000 µg/L amitriptyline did not allow a statistical comparison with the control (Table S8). In the second experiment, the exposure to amitriptyline as well as MP and the mixtures did not affect the AChE activity of brown trout (Table 4; Figure 4B; *F* = 0.3154, *df* = 5/174, *p* = 0.903). Amitriptyline and MP had no significant influence on the activity of the two tested CbEs (Table 3 and Table 4; first experiment: CbE-NPA *F* = 0.6629, *df* = 3/8.0916, *p* = 0.598, CbE-NPV *F* = 1.6918, *df* = 3/8.0474, *p* = 0.245; second experiment: CbE-NPA *F* = 1.6001, *df* = 5/173, *p* = 0.163, CbE-NPV *F* = 1.8906, *df* = 5/174, *p* = 0.098).

Neither amitriptyline nor MP or the mixture of both influenced the activity of SOD, the degree of LPO or the cortisol level (Table 3 and Table 4; first experiment: SOD *F* = 0.4596, *df* = 3/9.7439, *p* = 0.717, LPO *Χ^2^* = 1.5698, *df* = 3, *p* = 0.666, cortisol *Χ^2^* = 5.3672, *df* = 3, *p* = 0.147; second experiment: SOD *F* = 0.0336, *df* = 5/174, *p* = 0.999, LPO *Χ^2^* = 4.081, *df* = 5, *p* = 0.538, cortisol *F* = 1.3774, *df* = 3/83, *p* = 0.255, effect of video tracking on cortisol level *F* = 0.7279, *df* = 1/83, *p* = 0.396).

## 4. Discussion

We investigated the effects of the tricyclic antidepressant amitriptyline, PS MP, and their interaction on juvenile brown trout in two experiments.

### 4.1. Amitriptyline Concentrations

The analyzed amitriptyline concentrations in both experiments were considerably lower than the targeted concentrations, often below the LoQ. A calibration with external standards was used for the analyses of the amitriptyline concentrations without the addition of an internal standard. The sample matrix did not affect the quantification in the aqueous samples (spiked water samples compared to pure analytical standard). Deviation of the nominal and measured amitriptyline concentrations already occurred in the stock solutions. No incomplete dissolution or precipitation were observed in the stock solutions; however, this cannot be excluded. Prior to the experiments, the tanks were filled with amitriptyline solutions in the corresponding concentrations to saturate sorptive surfaces to reduce losses of the antidepressant by sorption. At the start of the experiments, the test media were exchanged. Since the discrepancy of nominal and measured concentrations occurred already at the beginning of the experiments, nonetheless sorption of amitriptyline to the glass walls of the bottles of the stock solution, vials used for sampling, and the aquaria or incomplete dissolution is likely. Lv et al. [74] and Chang et al. [75] observed an instantaneous uptake of a significant amount of amitriptyline to kaolinite and Ca-montmorillonite, respectively. Furthermore, amitriptyline was shown to adsorb considerably to polyamide (PA) MP [76]. Baena-Nogueras et al. [77] investigated the degradation kinetics of pharmaceuticals and found hydrolysis and biodegradation of amitriptyline negligible. Furthermore, in our experiment, photolysis of amitriptyline was unlikely due to the chosen illumination [77]. Uptake and metabolization of test media by trout can also be considered as a reason for the reduction of the amitriptyline concentrations in the water phase during the experiments. Bioaccumulation of amitriptyline was reported for several fish species [78,79,80,81,82]. Amitriptyline was shown to bioaccumulate in the brain, the target organ of the pharmaceutical, as well as gills, liver, plasma, bile, and to a low extent in the muscle. In addition, Ziarrusta et al. [82] identified 33 possibly toxicologically relevant metabolites of amitriptyline in gilt-head bream (*Sparus aurata*) after exposure to the antidepressant. Due to financial restrictions, residue analyses in fish were not included in our study. However, since the low amitriptyline concentrations already caused severe effects in the exposed fish, the aims of our study to investigate the effects of the antidepressant and the possible modulation of these effects by co-exposure of amitriptyline and PS MP were not affected. Due to uncertainties concerning the amitriptyline concentrations in our experiments, the lowest observed effect concentration (LOEC) could not be determined.

### 4.2. Mortality

Concentrations of 300 µg/L and 1000 µg/L amitriptyline (measured concentrations: 20 µg/L and 135 µg/L) caused severe reactions, which made it necessary to terminate the affected treatment group earlier than the other exposure groups. No other apparent changes in mortality were caused by amitriptyline, MP, or the mixtures of both which is in line with the results of Schmieg et al. [27]. Effects on the survival caused by exposure to high amitriptyline concentrations have been shown, e.g., for zebrafish (LC_50_ = 1.4 mg/L, 120 h; 3 mg/L, 144 h) and common carp (500 µg/L, 30 d) [28,29,30]. One explanation for the rapidly impaired condition of brown trout exposed to 1000 µg/L might be the serotonin syndrome, since high doses of tricyclic antidepressants are known to potentially cause this life-threatening condition in humans [24,83]. In contrast, the effects observed in the other treatment groups in our experiments increased over the exposure time. This might reflect the often-occurring delay of two weeks or longer until antidepressants have a therapeutic effect in humans [84].

### 4.3. Behavior during Exposure

Antidepressants are designed to alter behavior in humans. Therefore, effects on the behavior of non-target organisms, which possess similar molecular targets for the respective drugs, are plausible. In the present study, nominal concentrations of 100 µg/L amitriptyline and higher caused ataxic movements such as side or upside-down swimming in brown trout. Similar effects on the behavior were reported for zebrafish exposed to 10 mg/L amitriptyline for 20 min [24]. In both of our experiments, amitriptyline exposure increased the percentage of fish that stayed in the upper half of the tank significantly. The effect was considerably more pronounced in the first than in the second experiment. In addition to the different ages of the fish and the lower measured amitriptyline concentrations in the second experiment, biological variability might explain the observed difference. Comparable to our results, zebrafish exposed for 20 min to 1 mg/L and 5 mg/L amitriptyline or 2 weeks to 10 µg/L or 50 µg/L amitriptyline spend more time in the top of a novel tank and showed a significantly reduced latency to enter the top half of a novel tank [24,25]. The co-exposure of fish to amitriptyline and 10^4^ particles/L PS MP did not alter this effect of amitriptyline, whereas co-exposure to amitriptyline and 10^5^ particles/L PS MP mitigated the effect. Anxiolytic substances have been shown to increase the time spent in the upper half of the aquaria in a novel tank experiment. Reduced anxiety leads to an increase in exploration behavior [85]. Furthermore, the preference for brighter compartments was described as antianxiety behavior in fish [86]. In our experiments, the behavioral observations were not performed in a novel tank test, but during the exposure. However, the water column and water surface were brighter and more illuminated than the dark bottom of the aquaria. Therefore, the higher percentage of fish swimming in the upper half of the tanks should be interpreted as an anxiolytic effect of the antidepressant.

MP alone did not result in any changes in behavior. In zebrafish and marine medaka (*Oryzias melastigma*), exposure to polyethylene (PE) MP and polyvinyl chloride (PVC) MP did also not influence the time the fish spent in the top zone of a novel tank [87].

### 4.4. Video Tracking of Swimming Behavior

In the first experiment, fish were less active starting with 10 µg/L amitriptyline. This became obvious for the distance moved, but also for the inactivity and medium velocity at the end of the video tracking. Both exposure to amitriptyline and co-exposure to amitriptyline and PS MP in the second experiment significantly altered the fish’s behavior: trout treated with amitriptyline covered significantly less distance, swam with a lower velocity, and the time of inactivity was increased. Differences in the behavior between the two experiments can be partly explained by biological variance. The conditions during the video recording are stressful for the trout due to bright illumination and the limited amount of water in the recording aquaria [88]. Therefore, the activity of the control group can be interpreted as a period of freezing behavior followed by a flight response. There are two main explanations for the reduced level of activity in amitriptyline-treated trout: either the treatment causes anxiety and thereby increased freezing bouts [85] or, more likely, the behavior reflects the anxiolytic and sedative effects of the antidepressant and reduces the flight reflex. In accordance with the results of the present study, reduced covered distance and swimming velocity caused by amitriptyline exposure have been reported for brown trout larvae [27] and for adult zebrafish [24,25]. Furthermore, amitriptyline is described to decrease the swimming activity of zebrafish larvae in different exposure scenarios [26] and reduce the swimming distance under dark conditions and at high concentrations of 3 mg/L amitriptyline under dark as well as under light conditions [28].

No effect of MP on the swimming behavior of fish was found in the present study. Likewise, in different fish species, no or only minimal MP effects on swimming, exploration, or foraging behavior as well as shoaling or aggressive behavior and the boldness of fish or their predation risk have been found [27,51,87,89,90,91,92,93,94,95]. However, in other studies effects of MP on risk-taking behavior [96], feeding behavior [97,98], social behavior [98,99,100], and post-exposure predatory performance [101,102] were reported. PS and PE MP were found to cause reduced velocity and range of movement [98,100,103] but in other cases, PS MP and high-density PE MP led to increased activity and swimming distance [96,104,105]. Behavior is a sensitive endpoint that is affected by many parameters and the chosen experimental design. This as well as the different fish species, different MP types, and particle concentrations can explain the different results in the articles mentioned above.

### 4.5. Biochemical Responses

None of the treatment groups caused any effect on the oxidative stress level of juvenile brown trout. Consistently, amitriptyline exposure did not affect the oxidative stress level of the early life stages of brown trout [27]. In contrast, influences of amitriptyline on the oxidative stress level were observed in common carp [30] and zebrafish [29,32]. The results show that oxidative stress responses induced by amitriptyline are species-specific. Moreover, it is likely that higher concentrations of amitriptyline cause oxidative stress in fish, while lower concentrations only cause moderate effects or even improve the antioxidant capacity.

Many studies investigated the possible effects of PS MP on different parameters indicating oxidative stress in fish with considerably varying results [27,41,42,51,59,95,104,106,107,108]. However, it remains unclear under which conditions exposure to PS MP causes oxidative stress in fish. The results of all these studies show that effects are not just related to one parameter such as the size of the particles, but interdependences must be more complex. Possible explanations for the apparently contradictory results besides the size and shape of the MP are different experimental conditions and fish species or additives in the used polymers.

The hormone cortisol is known to be increased in reaction to stress in fish [109], and antidepressants were shown to reduce their cortisol level after handling stress [110]. In our experiment, the cortisol level of brown trout was not influenced by exposure to amitriptyline, PS MP, or the mixture. Consistent with the results of our experiment, Jakubowska et al. [111] found no significant effect of PS, polyethylene terephthalate (PET), or PE MP on sea trout (*Salmo trutta* f. *trutta*). Likewise, no effect on the cortisol level was found in juvenile brown trout after exposure to up to 1000 µg/L citalopram or 100 µg/L venlafaxine, two antidepressants [88,112].

Amitriptyline can bind to muscarinic acetylcholine receptors and was shown to reduce the AChE activity in the human erythrocyte membrane and serum [7,113]. This mode of action might be the mechanistic explanation for AChE activity being affected by amitriptyline in brown trout in the present study: 100 µg/L amitriptyline caused a significant increase in AChE while the activity of the two tested CbEs was slightly but not significantly decreased in the first experiment. However, in the second experiment, no significant effect of amitriptyline on the activity of AChE and CbEs was found. The lower amitriptyline concentrations in the second experiment might explain why no influence on AChE activity was observed. Accordingly, brown trout larvae exposed to higher amitriptyline concentrations (~48 µg/L) had a significantly higher activity of AChE and a significantly decreased activity of CbEs [27]. Furthermore, the activity of AChE was in general higher in the two months older trout of the second experiment, which could also indicate different sensitivities to neurotoxic effects at different life stages.

PS MP had no influence on the AChE or CbE activity. These results are consistent with other studies investigating effects of PS MP in juvenile and early life stages of brown trout [27,59], zebrafish larvae [51] or goldfish (*Carassius auratus*) [95]. In red tilapia (*Oreochromis niloticusas*) exposure to different sizes of PS MP caused either an increase or decrease in AChE activity after different times of exposure [106]. Moreover, Huang et al. [107] reported that AChE activity in the brain of red tilapia was significantly reduced after a 14 d treatment with 5 µm PS MP.

### 4.6. Co-Exposure of Amitriptyline and MP

After co-exposure to amitriptyline and PS MP, effects observed in fish did not differ much from those which occurred in fish exposed to amitriptyline alone. Only the highest concentration of MP slightly reduced the influence of the antidepressant on the swimming behavior during the experiment. One possible reason for this could be that the high MP concentration reduced the bioavailability of amitriptyline in the water. Wagstaff et al. [76] showed that significant adsorption of amitriptyline to PA MP occurs within 24 h at 20 °C (MP-wastewater distribution coefficient 749 L/kg).

Generally, it was shown in the past that different concentrations of MP can modulate the toxicity of chemicals in different ways. In larvae of marine medaka, the co-exposure to phenanthrene and PS MP alleviated the teratogenicity and lethality of phenanthrene [52]. However, the effect did not occur at higher MP concentrations and the phenanthrene concentration in the water was not changed [52]. A similar effect was observed by Chen et al. [51]: co-exposure of zebrafish to PS MP and low concentrations of 17α-Ethinylestradiol (EE2) reduced the effects of EE2 on the locomotion of the fish. In contrast, mixtures of PS MP with higher EE2 concentrations even enhanced the effect of EE2 on the locomotion of zebrafish [51]. The study of Huang et al. [107] showed complex interactions of the co-exposure to virgin or altered PS MP with two pharmaceuticals in red tilapia. For example, the inhibitory effect on AChE activity caused by the antibiotic sulfamethoxazole was alleviated by co-exposure to aged PS MP [107]. In these studies, the authors hypothesized that the described effects were caused by a reduction of bioavailability of the tested substances due to adsorption to the MP [51,52,107].

## 5. Conclusions

The highest tested amitriptyline concentration of 1000 µg/L (measured concentration 135 µg/L) caused life-threatening conditions in brown trout within one day and significant behavioral changes occurred at low amitriptyline concentrations in the magnitude of µg/L. To assess the risk of the antidepressant, mixture toxicity should also be taken into consideration: most of the used pharmaceuticals to treat depression share the same mode of action and increase the serotonin, noradrenaline, and dopamine concentrations in the synaptic cleft. Therefore, it can be anticipated that if trout are exposed to different antidepressants, the effects of the substances affect the organisms in an additive way.

In contrast, PS MP did not influence any of the investigated parameters at high concentrations of 10^4^ and 10^5^ particles/L. Furthermore, MP did not modulate most of the effects caused by amitriptyline exposure. However, the effects of MP on fish in general and possible modulations by MP of effects resulting from other pollutants should not be underestimated and underlying mechanisms should be further investigated.

## Figures and Tables

**Table 1:** Nominal and measured amitriptyline concentrations of the first experiment (limit of quantification (LoQ) 0.1 µg/L; n.a.: not analyzed; for calculation of mean exposure concentrations values below the limit of quantification were substituted with LoQ/√2).

Nominal Concentration [µg/L]	Start of the Experiment [µg/L]	Before Water Exchange [µg/L]	After Water Exchange [µg/L]	End of the Experiment [µg/L]	Calculated Mean of Measured Concentration [µg/L]
0	<0.1	<0.1	<0.1	<0.1	0
1	0.10	<0.1	<0.1	<0.1	0.08
10	1.25	0.45	0.71	0.82	0.8
100	48.90	<0.1	<0.1	<0.1	12
300	25.00	12.48	22.97	n.a.	20
1000	135.18	n.a.	n.a.	n.a.	135

**Table 2:** Measured amitriptyline concentrations of the second experiment (limit of quantification (LoQ) 0.1 µg/L; for calculation of mean exposure concentrations values below the limit of quantification were substituted with LoQ/√2; MP_tt_: 10^4^ particles/L PS MP; MP_ht_: 10^5^ particles/L PS MP; MIX_tt_: amitriptyline + 10^4^ particles/L; MIX_ht_: amitriptyline + 10^5^ particles/L).

Abbreviation Treatment Group	Nominal Concentration [µg/L]	Start of the Experiment [µg/L]	Before Water Exchange [µg/L]	After Water Exchange [µg/L]	End of the Experiment [µg/L]	Calculated Mean of Measured Concentration [µg/L]
control	0	<0.1	<0.1	<0.1	<0.1	0
amitriptyline	100	<0.1	<0.1	<0.1	0.18	0.10
MP_tt_	0	<0.1	<0.1	<0.1	<0.1	0
MP_ht_	0	<0.1	<0.1	<0.1	<0.1	0
MIX_tt_	100	<0.1	<0.1	5.73	0.13	1.50
MIX_ht_	100	5.52	<0.1	<0.1	0.59	1.56

**Table 3:** Results of the first experiment. If significant differences occurred, *p* values compared to the control group are mentioned. Significant differences to the control group are indicated with an asterisk and highlighted in bold.

	Control	1 µg/L Amitriptyline	10 µg/L Amitriptyline	100 µg/L Amitriptyline
Mortality (%)	17 ± 12	20 ± 14	13 ± 19	20 ± 0
Length (cm)	6.1 ± 0.4	6.1 ± 0.6	6.1 ± 0.8	6.1 ± 0.6
Body mass (g)	2.22 ± 0.37	2.31 ± 0.68	2.41 ± 0.83	2.01 ± 0.63
Fish in upper half of the tank (%)	1 ± 4	1 ± 2 *p* = 0.671	1 ± 3 *p* = 0.814	**37 ± 25** * p < 0.001
Total distance moved (cm)	1101 ± 528	1864 ± 1039 *p* = 0.561	**486** **± 615** * *p* = 0.027	786 ± 652 *p* = 0.577
Mean velocity (cm/s)	1.02 ± 0.49	1.73 ± 0.96 *p* = 1	**0.45 ± 0.57** * *p* = 0.009 **(end of recording)**	**0.73 ± 0.60** * *p* = 0.045 **(end of recording)**
No movement (s)	892 ± 116	721 ± 216 *p* = 0.862	**1010 ± 133** * *p* = 0.001 **(end of recording)**	**954 ± 153** * *p* = 0.006 **(end of recording)**
Lipid peroxidation (CHP-equiv.)	28.80 ± 13.79	29.86 ± 12.73	27.10 ± 13.34	30.48 ± 15.77
SOD (U/mL)	85.69 ± 30.99	93.13 ± 29.27	86.70 ± 28.07	84.32 ± 27.95
Cortisol level (ng/mL)	11.53 ± 21.13	16.16 ± 23.26	20.19 ± 32.72	28.16 ± 39.20
AChE activity (mu/mg protein)	96.53 ± 20.66	94.77 ± 28.28 *p* = 0.946	86.41 ± 21.45 *p* = 0.361	**120.35 ± 33.37** * *p* = 0.033
CbE-NPA activity (mu/mg protein)	130.84 ± 23.38	122.71 ± 30.09	107.52 ± 24.60	116.78 ± 32.42
CbE-NPV activity (mu/mg protein)	98.24 ± 22.13	88.98 ± 27.04	70.29 ± 22.29	79.88 ± 32.18

**Table 4:** Summary of the results of the second experiment (MP_tt_: 10^4^ particles/L PS MP; MP_ht_: 10^5^ particles/L PS MP; MIX_tt_: amitriptyline + 10^4^ particles/L; MIX_ht_: amitriptyline + 10^5^ particles/L; n.a.: not analyzed). If significant differences occurred *p* values compared to the control group are mentioned. Significant differences to the control group are indicated with an asterisk and highlighted in bold. For significant differences between the treatment groups see Table S7.

	Control	Amitriptyline	MP_tt_	MP_ht_	MIX_tt_	MIX_ht_
Mortality	0 ± 0	0 ± 0	0 ± 0	0 ± 0	0 ± 0	0 ± 0
Length (cm)	7.0 ± 0.6	6.9 ± 0.6	6.9 ± 0.6	7.2 ± 0.6	6.8 ± 0.7	7.2 ± 0.8
Body mass (g)	3.09 ± 0.75	3.14 ± 1.43	3.02 ± 0.81	3.39 ± 0.87	2.88 ± 0.78	3.31 ± 1.00
Fish in upper half of the tank (%)	0 ± 0	**8 ± 10** * *p* = 0.009	0 ± 0 *p* = 1	0 ± 0 *p* = 1	**8 ± 11** * *p* = 0.006	2 ± 6 *p* = 0.862
Total distance moved (cm)	1733 ± 1134	**609 ± 677** * *p* = 0.034	1746 ± 1021 *p* = 1	1506 ± 780 *p* = 0.9997	**549 ± 579** * *p* = 0.014	**240 ± 217** * *p* < 0.001
Mean velocity (cm/s)	1.60 ± 1.05	**0.56 ± 0.63** * *p* = 0.035	1.62 ± 0.95 *p* = 0.993	1.39 ± 0.72 *p* = 0.958	**0.51 ± 054** * *p* = 0.014	**0.22 ± 0.20** * *p* < 0.001
No movement (s)	752 ± 246	**1000 ± 155** * *p* = 0.105	742 ± 222 *p* = 0.992	798 ± 157 *p* = 0.943	**999 ± 128** * *p* = 0.012	**1062 ± 28** * *p* < 0.001
Lipid peroxidation (CHP-equiv.)	23.36 ± 8.78	23.93 ± 9.15	27.33 ± 7.54	25.06 ± 9.15	26.18 ± 10.21	22.49 ± 8.76
SOD (U/mL)	109.62 ± 37.39	109.42 ± 37.43	110.01 ± 34.91	108.05 ± 37.18	112.48 ± 42.76	108.96 ± 40.70
Cortisol level (ng/mL)	40.60 ± 31.25	41.36 ± 29.15	n.a.	34.21 ± 35.77	n.a.	50.93 ± 31.63
AChE activity (mu/mg protein)	122.73 ± 39.06	131.47 ± 44.14	134.05 ± 54.36	122.59 ± 26.03	132.23 ± 38.72	127.52 ± 49.12
CbE-NPA activity (mu/mg protein)	87.59 ± 15.15	86.82 ± 27.89	93.26 ± 25.70	84.72 ± 15.29	90.74 ± 22.89	78.97 ± 15.67
CbE-NPV activity (mu/mg protein)	50.66 ± 12.25	50.74 ± 23.55	59.35 ± 21.81	54.00 ± 21.00	54.47 ± 17.62	45.92 ± 15.52

## Data Availability

Data are contained within the article or Supplementary Materials.

## Supplementary Materials

The following supporting information can be downloaded at: https://www.mdpi.com/article/10.3390/toxics10120763/s1, Table S1: Size ranges and counted particle numbers of PS particles in an exemplarily measured stock suspension; Figure S1: Histogram of size ranges and counted particle numbers of PS MP in an exemplarily measured stock suspension; Table S2: Water parameters first experiment; Table S3: Water parameters second experiment; Table S4: Operating parameters of the triple quadrupole MS (Agilent 6490 QqQ) in positive mode (ESI+); Table S5: Specific measurement parameters for amitriptyline with LC-QqQ in water samples. Intraday variation (RSD) is calculated with a 1 μg/L standard (10 μL injection volume and 4 replicates); Table S6: Used data transformations; Table S7: Further statistical information on the second experiment; Table S8: Results of the treatment groups with 300 µg/L and 1000 µg/L amitriptyline; CRED Reporting; Data first experiment; Data second experiment.
