# Supplementary material for: Effects of the Antidepressant Amitriptyline on Juvenile Brown Trout and Their Modulation by Microplastics
Source: Toxics. 2022 Dec 7;10(12):763. doi: 10.3390/toxics10120763 (PMC9787892; doi:10.3390/toxics10120763)
Supplement: Supplementary file 1 [file toxics-10-00763-s001.zip › Supplement_Schmieg et al. Effects of the Antidepressant Amitriptyline on Juvenile Brown Trout and their Modulation by Microplastics.pdf]

Supplementary Materials

Table S1. Size ranges (in μm) and counted particle numbers (per mL) of polystyrene particles in an exemplarily measured stock suspension.

Size range (μm)	Particle number (1/mL)
2-3	9067
3-4	6488
4-5	8872
5-6	4832
6-7	3770
7-8	3484
8-9	3076
9-10	2412
10-12	3259
12-14	2330
14-16	1628
16-18	1195
18-20	1081
20-25	1698
25-30	1041
30-35	643
35-40	473
40-50	511
50-60	220
60-70	89
70-80	43
80-90	18
90-100	6
100-150	5
150-200	0
200-250	0
250-300	0
300-350	0
350-400	0

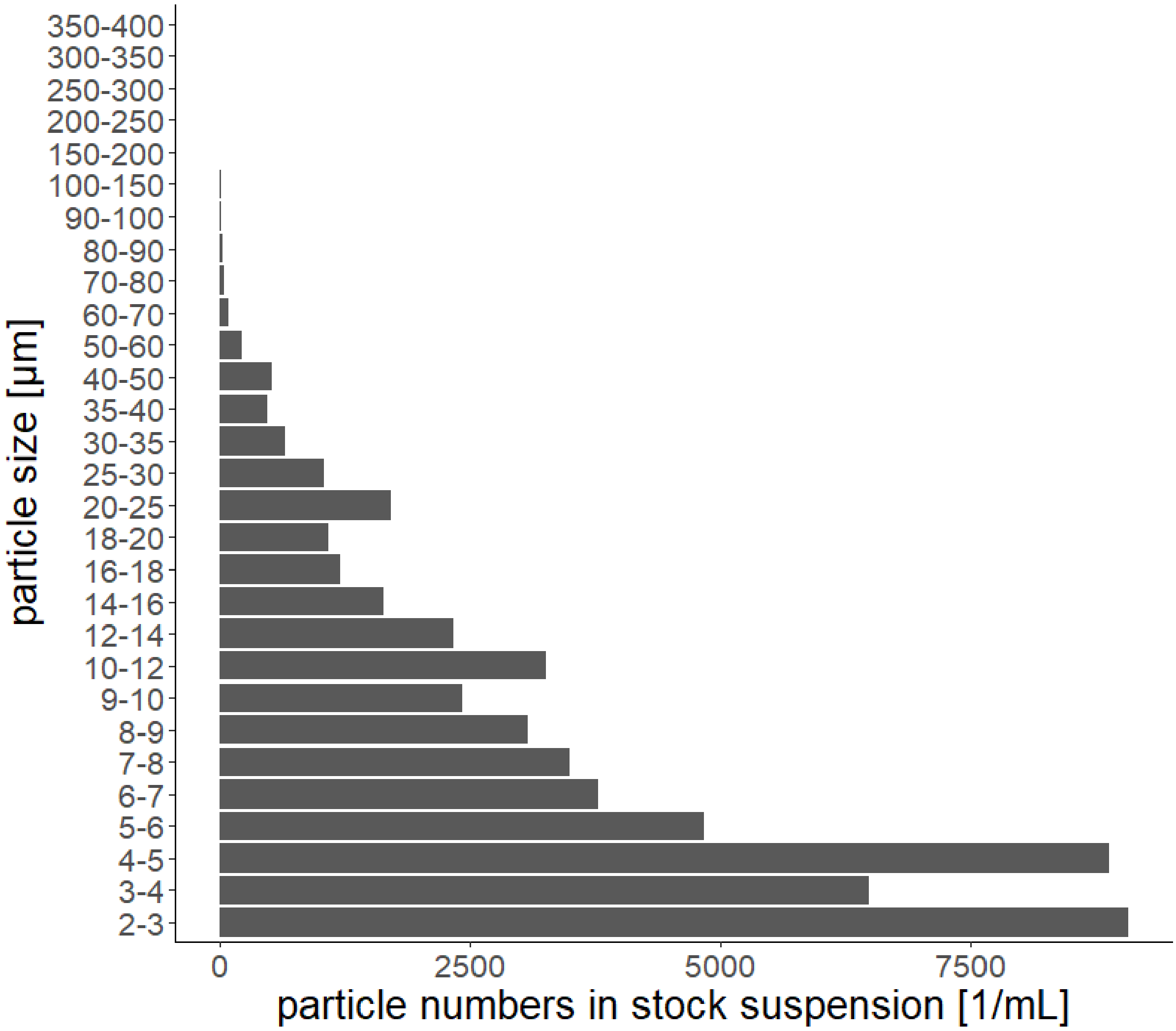

Figure S1. Size ranges and counted particle numbers of polystyrene particles in an exemplarily measured stock suspension.

Table S2. Water parameters first experiment (n.a.: not analyzed).

Start of experiment							
**Block**	**Treatment (amitriptyline concentration)**	**pH**	**Temperature (°C)**	**Conductivity (µS/cm)**	**O_2 (%)**	**O_2 (mg/L)**	**Nitrite (mg/L)**
1	control	7.53	6.8	479	11.13	95.8	n.a.
1	1 µg/L	7.44	6.5	484	11.26	96.1	n.a.
1	10 µg/L	7.52	7	482	11.13	96.2	n.a.
1	100 µg/L	7.69	7	487	11.06	95.6	n.a.
1	300 µg/L	7.69	6.4	485	11.28	96	n.a.
1	1000 µg/L	7.82	7.1	483	10.86	94.2	n.a.
2	control	7.45	6.5	480	11.11	94.8	n.a.
2	1 µg/L	7.47	6.1	484	11.39	96.3	n.a.
2	10 µg/L	7.57	6.4	480	11.1	94.6	n.a.
2	100 µg/L	7.67	6.8	488	11.06	95.2	n.a.
2	300 µg/L	7.73	6.2	484	11.31	96	n.a.
2	1000 µg/L	7.81	6.5	483	11.12	94.9	n.a.

3	control	7.42	6.5	481	11.33	96.7	n.a.
3	1 µg/L	7.48	6.9	486	11.18	96.5	n.a.
3	10 µg/L	7.62	6.4	480	11.29	96.2	n.a.
3	100 µg/L	7.64	6.5	484	11.34	96.8	n.a.
3	300 µg/L	7.74	6.7	484	11.26	96.6	n.a.
3	1000 µg/L	7.79	6.4	482	11.16	95	n.a.
**End of experiment**							
**Block**	**Treatment (amitriptyline concentration)**	**pH**	**Temperature (°C)**	**Conductivity (µS/cm)**	**O_2 (%)**	**O_2 (mg/L)**	**Nitrite (mg/L)**
1	control	7.21	6.9	482	11.18	96.5	0.1
1	1 µg/L	7.23	6.8	481	11.21	96.5	0.1
1	10 µg/L	7.31	6.7	477	11.26	96.7	0.1
1	100 µg/L	7.31	6.8	479	11.23	96.6	0.1
1	300 µg/L	n.a.	n.a.	n.a.	n.a.	n.a.	n.a.
1	1000 µg/L	n.a.	n.a.	n.a.	n.a.	n.a.	n.a.
2	control	7.24	6.8	482	11.03	95.5	0.1
2	1 µg/L	7.23	6.4	480	11.36	96.7	0.1
2	10 µg/L	7.29	6.3	477	11.38	96.7	0.1
2	100 µg/L	7.33	6.6	479	11.31	96.8	0.1
2	300 µg/L	n.a.	n.a.	n.a.	n.a.	n.a.	n.a.
2	1000 µg/L	n.a.	n.a.	n.a.	n.a.	n.a.	n.a.
3	control	7.28	6.8	480	11.13	95.9	0.1
3	1 µg/L	7.25	6.4	479	11.31	96.5	0.1
3	10 µg/L	7.27	6.3	475	11.34	96.5	0.1
3	100 µg/L	7.35	6.5	477	11.29	96.3	0.1
3	300 µg/L	n.a.	n.a.	n.a.	n.a.	n.a.	n.a.
3	1000 µg/L	n.a.	n.a.	n.a.	n.a.	n.a.	n.a.

Table S3. Water parameters second experiment (n.a.: not analyzed).

Block	Treatment	pH	Temperature (°C)	Conductivity (µS/cm)	O_2 (%)	O_2 (mg/L)	Nitrite (mg/L)	Total hardness
**Start of experiment**								
1	control	7.98	7.0	476	10.92	94.2	n.a.	n.a.
1	MP_{tt}	7.83	6.7	477	10.91	93.4	n.a.	n.a.
1	MP_{ht}	7.77	6.6	476	10.98	93.8	n.a.	n.a.
1	amitriptyline	7.66	6.8	474	11.05	94.7	n.a.	n.a.
1	MIX_{tt}	7.66	6.6	475	11.15	95.3	n.a.	n.a.
1	MIX_{ht}	7.65	6.5	475	11.02	94	n.a.	n.a.
2	control	7.90	6.6	474	10.83	92.6	n.a.	n.a.
2	MP_{tt}	7.82	6.5	476	10.98	93.5	n.a.	n.a.
2	MP_{ht}	7.74	6.5	477	10.69	91.2	n.a.	n.a.
2	amitriptyline	7.66	6.6	475	10.91	92.9	n.a.	n.a.
2	MIX_{tt}	7.66	6.4	476	11.02	93.7	n.a.	n.a.
2	MIX_{ht}	7.66	6.3	474	11.03	93.5	n.a.	n.a.
3	control	7.84	6.6	477	10.81	92.3	n.a.	n.a.
3	MP_{tt}	7.78	6.6	478	10.62	90.8	n.a.	n.a.
3	MP_{ht}	7.73	6.5	477	10.52	89.7	n.a.	n.a.
3	amitriptyline	7.66	6.4	474	10.96	93.2	n.a.	n.a.
3	MIX_{tt}	7.66	6.5	477	10.86	92.5	n.a.	n.a.
3	MIX_{ht}	7.65	6.3	476	10.7	90.7	n.a.	n.a.
**End of experiment**								
**Block**	**Treatment**	**pH**	**Temperature (°C)**	**Conductivity (µS/cm)**	**O_2 (%)**	**O_2 (mg/L)**	**Nitrite (mg/L)**	**Total hardness**
1	control	7.62	6.6	486	11.41	97.1	0,05 - 0,1	16
1	MP_{tt}	7.41	6.3	487	11.5	97.4	0,05 - 0,1	14
1	MP_{ht}	7.42	6.3	486	11.49	97.3	0,05 - 0,1	15
1	amitriptyline	7.48	6.2	486	11.47	96.8	0.1	15
1	MIX_{tt}	7.53	6.1	486	11.49	96.8	0,05 - 0,1	15
1	MIX_{ht}	7.56	6.3	485	11.46	96.9	0,05 - 0,1	15
2	control	7.57	6.3	486	11.35	96	0,05 - 0,1	16
2	MP_{tt}	7.41	6.2	483	11.49	96.9	0.1	15
2	MP_{ht}	7.45	6.3	485	11.42	96.6	0.1	15
2	amitriptyline	7.5	6.2	484	11.48	96.9	0.1	15
2	MIX_{tt}	7.54	6.1	482	11.42	96.2	0.1	15
2	MIX_{ht}	7.58	6	480	11.41	95.9	0.1	14
3	control	7.57	6.2	483	11.47	96.8	0.1	15
3	MP_{tt}	7.51	6.3	486	11.48	97.2	0.1	16
3	MP_{ht}	7.46	6.2	482	11.43	96.5	0.1	15
3	amitriptyline	7.51	6.1	484	11.39	95.9	0.1	15
3	MIX_{tt}	7.55	6.2	488	11.36	96	0,05 - 0,1	15
3	MIX_{ht}	7.58	6	485	11.52	96.8	0,05 - 0,1	15

Table S4. Operating parameters of the triple quadrupole MS (Agilent 6490 QqQ) in positive mode (ESI +).

**Instrument Parameters**	
Gas Temp (°C)	150
Gas Flow (L/min)	16
Nebulizer (psig)	40
Sheath gas temperature (°C)	300
Sheath gas flow	12
**Source Parameters**	
Capillary voltage (V)	4200
Nozzle voltage (V)	1000

Table S5. Specific measurement parameters for amitriptyline with LC-QqQ in water samples. Intraday variation (RSD) is calculated with a 1 µg/L standard (10 µl injection volume and 4 replicates); FV: fragmentor voltage; RT: retention time; LOQ: Limit of quantification.

**Precursor Ion**	**Product Ion 1 (CE in eV)**	**Product Ion 2 (CE in eV)**	**FV (V)**	**RT (min)**	**RSD (%) n = 4**	**LOQ (ng/L)**
278.2	117 (21)	91 (25)	380	5.2	2	100

Table S6. Used data transformations.

**Experiment**	**Endpoint**	**Transformation**
1	AChE	log(x)
1	CbE-NPA	sqrt (x)
1	CbE-NPV	sqrt (x)
1	SOD	sqrt (x)
1	Mean velocity	log(x) (variance of data part of effect)
1	Mean velocity (end of recording)	log (x)
1	Time of no movement	x^3 (variance of data part of effect)
1	Total distance covered	log(x)
2	weight	log (x)
2	length	x^2
2	AChE	log(x)
2	CbE-NPA	1/x
2	CbE-NPV	1/sqrt(x)
2	SOD	sqrt (x)
2	Cortisol	sqrt (x)
2	Mean velocity	sqrt (x) (variance of data part of effect)
2	Time of no movement	x^3 (variance of data part of effect)
2	Total distance covered	sqrt(x)

Table S7. Further statistical information on the second experiment. Significant differences are indicated with an asterisk and highlighted in bold. Trends are highlighted italic.

	Group 1	Group 2	*p* value
Fish in upper part of the tank	**Control**	**Amitriptyline**	**0.009***
	Control	MP_{tt}	1.000
	Control	MP_{ht}	1.000
	**Control**	**MIX_{tt}**	**0.006***
	Control	MIX_{ht}	0.862
	Amitriptyline	MIX_{tt}	1.000
	Amitriptyline	MIX_{ht}	0.190
	MP_{tt}	MP_{ht}	1.000
	**MP_{tt}**	**MIX_{tt}**	**0.006***
	MP_{ht}	MIX_{ht}	0.862
	**Group 1**	**Group 2**	***p* value**
Mean velocity	**Control**	**Amitriptyline**	**0.035***
	Control	MP_{tt}	0.993
	Control	MP_{ht}	0.958
	**Control**	**MIX_{tt}**	**0.014***
	**Control**	**MIX_{ht}**	**<0.001***
	Amitriptyline	MIX_{tt}	1.000
	Amitriptyline	MIX_{ht}	0.691
	MP_{tt}	MP_{ht}	1.000
	MP_{tt}	*MIX_{tt}*	*0.0677 TREND*
	**MP_{ht}**	**MIX_{ht}**	**0.005***
	**Group 1**	**Group 2**	***p* value**
Time of no movement	**Control**	**Amitriptyline**	**0.011***
	Control	MP_{tt}	0.992
	Control	MP_{ht}	0.943
	**Control**	**MIX_{tt}**	**0.012***
	**Control**	**MIX_{ht}**	**<0.001***
	Amitriptyline	MIX_{tt}	1.000
	Amitriptyline	MIX_{ht}	0.940
	MP_{tt}	MP_{ht}	0.999
	MP_{tt}	*MIX_{tt}*	*0.066 TREND*
	**MP_{ht}**	**MIX_{ht}**	**0.012***
	**Group 1**	**Group 2**	***p* value**
Total distance moved	**Control**	**Amitriptyline**	**0.034***
	Control	MP_{tt}	1.000
	Control	MP_{ht}	1.000
	**Control**	**MIX_{tt}**	**0.014***
	**Control**	**MIX_{ht}**	**<0.001***
	Amitriptyline	MIX_{tt}	1.000
	Amitriptyline	MIX_{ht}	0.679
	MP_{tt}	MP_{ht}	0.998
	**MP_{tt}**	**MIX_{tt}**	**0.009***
	**MP_{ht}**	**MIX_{ht}**	**<0.001***

Table S8. Results of the treatment groups with 300 µg/L and 1000 µg/L amitritpyline which were terminated after 7d and 1 d, respectivly.

	**300 µg/L amitriptyline**	**1000 µg/L amitriptyline**
**Mortality (%)**	n.a.	n.a.
**Length (cm)**	6.0 ± 0.7	6.3 ± 0.7
**Body mass (g)**	1.98 ± 0.73	2.36 ± 0.83
**Fish in upper half of the tank (%)**	n.a.	n.a.
**Total distance moved (cm)**	n.a.	n.a.
**Mean velocity (cm/s)**	n.a.	n.a.
**No movement (s)**	n.a.	n.a.
**Lipid peroxidation (CHP-equiv.)**	28.26 ± 11.62	26.74 ± 13.10
**SOD (U/mL)**	84.01 ± 29.36	86.56 ± 36.73
**Cortisol level (ng/mL)**	40.35 ± 50.72	46.91 ± 54.50
**AChE activity (mu/mg protein)**	122.60 ± 29.92	110.22 ± 24.17
**CbE-NPA activity (mu/mg protein)**	103.44 ± 23.03	92.31 ± 19.85
**CbE-NPV activity (mu/mg protein)**	65.45 ± 26.24	58.26 ± 22.50

CRED Reporting

**1. General information**	
a) Purpose of study	Evaluation of effects of polystyrene microplastic particles, the antidepressant amitriptyline, and the mixture of both on health and behavior of juvenile brown trout
b) Description of endpoints	Mortality, biometric values, behavior during the experiment, behavior in a stressful environment, level of lipid peroxides (FOX), SOD activity, AChE activity, CbE activity, tissue cortisol concentration
**2. Test design**	
a) standard/modified standard	Not performed according to standard procedures
b) Good Laboratory Practices (GLP)	Not GLP-accredited
c) Controls	Negative control (Per experiment 30 fish separated in 3 replicate tanks)
d) Validity	First experiment: Control mortality < 20%, oxygen concentration was over 60%, and the temperature difference between the aquaria was smaller than 1.5 °C<br>Second experiment: Control mortality 0%, oxygen concentration was over 60% and the temperature difference between the aquaria was smaller than 1.5 °C
**3.1. Test compound – amitriptyline**	
a) Identification	Amitriptyline hydrochloride (CAS number: 549-18-8; Molecular Formula: $C_{20}H_{23}N \cdot HCl$; molecular weight 313.86)
b) Physico-chemical characteristics	Octanol/water partition coefficient: log Pow 5.0; melting point; 198 -200 °C
c) Source	Sigma-Aldrich, Lot: BCBV1175
d) Purity in %	≥98 %;
e) Formulation	No formulation, no impurities
**3.2. Test compound – polystyrene**	
a) Identification	Polystyrene pellets (Polystyrol 158 K)
b) Physico-chemical characteristics	Transparent, density: 1.05 g/mL
c) Source	BASF, Germany
**4. Test organism**	
a) Scientific name	*Salmo trutta* f. *fario*
b) Body weight/length	First experiment: 2.2 ± 0.67 g / 6.1 ± 0.59 cm<br>Second experiment: 3.1 ± 0.86 g / 7.0 ± 0.68 cm
c) Age/life stage	Juvenile
d) Reproductive condition	Not in reproductive condition
e) Sex	Not determinable
f) Strain/clone	No defined clone
g) Source	Commercial trout farm (Forellenzucht Lohmühle, Alprisbach-Ehlenbogen, Germany)
h) Acclimatization	First experiment: 1 week<br>Second experiment: 9 weeks
**5. Exposure conditions**	

a) Schedule	Semi-static system; 50% water exchanges twice a week
b) System	Closed
c) Test medium	Filtered tap water (iron filter, active charcoal filter, particle filter) cooled and aerated
d) Temperature	Climate chamber set to 7 °C; first experiment: 6.60 ± 0.25 °C; second experiment: 6.39 ± 0.23 °C
e) pH	First experiment: average pH 7.5 ± 0.2<br>Second experiment: average pH 7.6 ± 0.1
f) Hardness	First experiment: not measured<br>Second experiment: total hardness 15 ± 0.5
g) Conductivity	First experiment: average conductivity 481.5 ± 3.1 µS/cm<br>Second experiment: average conductivity 480.2 ± 4.7 µS/cm
h) Dissolved oxygen	First experiment: 11.21 ± 0.12 mg/L, 96.02 ± 0.72%<br>Second experiment: 11.17 ± 0.30 mg/L, 94.78 ± 2.17%
i) Light intensity/quality	10 h/14 h light/dark cycle; tanks were shaded from direct light with black plastic foil
j) Feeding	Daily 3% of body weight of commercially available trout feed (0.8 mm, Incio Plus, Biomar, Brande, Denmark)
k) Aquaria	20 L glass aquaria filled with 15 L of medium, covered with glass plane, silicone tubing and glass pipette for aeration
l) Sand/sediment	No sediment tested
m) Stock solutions	For the first experiment two stock solutions were prepared with bi-distilled water: stock solution I contained 100 mg/L amitriptyline. For stock solution II, with a concentration of 1 mg/L, 10 mL of stock solution I were diluted with 990 mL bi-distilled water. For the second a stock solution with a concentration of 25 mg/L amitriptyline was prepared.<br><br>For polystyrene: 56,240 particles/mL
n) Nominal concentrations	First experiment: 0, 1, 10, 100, 300, 1000 µg/L amitriptyline<br>Second experiment: negative control, 100 µL/L amitriptyline, 10^4 particles/L PS MP, 10^5 particles/L PS MP and the mixtures 100 µg/L amitriptyline + 10^4 particles/L and 100 µg/L amitriptyline + 10^5 particles/L
o) Measured concentrations	See Table 1 and Table 2
p) Method	Liquid chromatography–mass spectrometry (LC-MS)
q) Duration	First experiment: 21/22 days (31.07.2018 - 21.8.2018/22.8.2018)<br>Second experiment: 21/22 days (27.09.2018-18/19.10.2018).

r) Observations	See material and methods
s) Results	See Table 3, Table 4 in the manuscript
t) Biomass loading	At the end of the experiments:<br>First experiment: 1.47 g/L<br>Second experiment: 2.07 g/L
**6. Statistical Design and Biological Response**	
a) Replicates	Three replicate aquaria per test concentration
b) Number of organisms	10 individual fish per replicate
c) Design	Three blocks, one replicate per treatment present in each block, placed randomized within each block
d) Statistical methods	Mortality: mixed effects Cox model (package coxme, treatment as fixed effect and aquarium ID as random effect; length, weight (second experiment), AChE, CbE-pnpa, CbE-pnpv, SOD, total distance moved, velocity at the last minute of recording (first experiment): linear mixed model (package lme4, treatment as fixed and aquarium ID as random effect); cortisol level (second experiment): linear mixed model (package lme4, treatment and "inclusion in video tracking" as fixed and aquarium ID as random effect); mean velocity and time of no movement: linear mixed model (package lme4, treatment and recording time as fixed and aquarium ID as random effect); Weight (first experiment): Welch-ANOVA; Cortisol (first experiment), time of no movement at last minute of recording (first experiment) and LPO: Kruskal Wallis test; position of the fish in the tank during the experiments: generalized linear mixed models (treatment group and days of exposure as fixed factors and aquarium ID as random factor; second experiment package glmmTMB). In the first experiment Dunnett's Test or Conover`s many-to one test was used as a post hoc test in the second experiment Tukey's all-pair comparisons. If necessary multiple comparisons were corrected with the method of Benjamini and Hochberg (1995).
e) Biological response	No effect on mortality, length, weight, SOD activity, LPO level, activity of CbEs and cortisol level in both experiments. No effect in first experiment on mean velocity (trend: decreased in 10 and 100 µg/L), time of no movement (trend: increased in 10 and 100 µg/L), No effect on AChE in the second experiment.<br>First experiment: Ataxic movements (300 µg/L and 1000 µg/L amitriptyline); More fish in upper half of the tank (100 µg/L amitriptyline); Total distance move decreased (10 µg/L amitriptyline); Reduced velocity and increased

	time of no movement at last minute of recording (10 and 100 µg/L amitriptyline); Increased AChE activity (100 µg/L amitriptyline) Second experiment: More fish in upper half of the tanks (amitriptyline and MIX_{tt}); Reduced mean velocity and distance moved (amitriptyline and MIX_{tt}, MIX_{ht}); increased time of no movement (amitriptyline and MIX_{tt}, MIX_{ht}).
f) Dose-response	First experiment: ataxic movements in fish exposed to highest tested concentrations (300 and 1000 µg/L amitriptyline). More fish in upper half of the tank during the experiment and increased AChE activity in the highest, for the whole experiment tested concentration (100 µg/L amitriptyline). Decreased velocity and increased inactivity at end of video tracking of two highest tested amitriptyline concentrations (10 and 100 µg/L amitriptyline). No dose response was observed for total distance moved as only a significant decrease occurred in fish exposed to 10 but not to 100 µg/L amitriptyline. Second experiment: not tested
g) Statistical significances	First experiment: More fish in upper half of tank (control – 100 µg/L amitriptyline), decreased total distance moved (control - 10 µg/L amitriptyline), reduced velocity and increased time of no movement at last minute of recording (control -10 µg/L amitriptyline and control-100 µg/L amitriptyline) and increased AChE activity (control - 100 µg/L amitriptyline). Second experiment: More fish in upper half of the tank during the experiment (control – amitriptyline and MIX_{tt}; MP_{tt}-MIX_{tt}), decreased distance moved (control - amitriptyline, MIX_{tt} and MIX_{ht}; MP_{tt}-MIX_{tt}; MP_{ht} – MIX_{ht}), mean velocity (control - amitriptyline, MIX_{tt} and MIX_{ht}; MP_{ht} – MIX_{ht}; Trend: MP_{tt}-MIX_{tt}), and increased time of no movement (control - amitriptyline, MIX_{tt} and MIX_{ht}; MP_{ht} – MIX_{ht}; Trend: MP_{tt}-MIX_{tt}).
h) Significance level	α= 0.05, in case of multiple comparisons adjusted via the method of Benjamini and Hochberg
i) Variability	Not estimated
j) Raw data	Provided in supplementary material
